# Reliability of Variables of Consonant Production and Velopharyngeal Competence in 10-year-olds with Cleft Palate Using Multiple Raters

**DOI:** 10.1177/10556656241287761

**Published:** 2024-10-03

**Authors:** Kristina Klintö, Malin Schaar Johansson, Magdalena Andersson, Caroline Gällstedt, Cecilia Lindberg, Cecilia Nelli, Åsa Okhiria

**Affiliations:** 1 Division of Speech Language Pathology, Phoniatrics and Audiology, Department of Clinical Sciences in Lund, 5193 Lund University, Lund, Sweden; 2 Division of Speech Language Pathology, Department of Otorhinolaryngology, 59565 Skåne University Hospital, Malmö, Sweden; 3 Division of Speech and Language Pathology, Department of Head-, Neck- and Plastic Surgery, 59566 Örebro University Hospital, Örebro, Sweden; 4 Speech and Language Pathology, Department of Clinical Sciences, 8075 Umeå University, Umeå, Sweden; 5 Speech and Language Pathology, Department of Otorhinolaryngology, 56749 Sahlgrenska University Hospital, Gothenburg, Sweden; 6 Speech and Language Pathology Unit, Otorhinolaryngology Clinic, 56750 University Hospital Linköping, Linköping, Sweden; 7 Medical Unit Speech and Language Pathology and Department of Plastic Surgery and Craniofacial Surgery, 59562 Karolinska University Hospital, Stockholm, Sweden

**Keywords:** cleft lip and palate, speech variables, quality indicators, reliability, quality registry

## Abstract

**Objective:**

To assess the reliability of speech data and speech-related quality indicators in the Swedish quality registry for cleft lip and palate (CLP) at 10 years of age.

**Design:**

Retrospective study.

**Setting:**

University hospitals.

**Participants:**

One hundred twenty-one 10-year-olds with unilateral or bilateral CLP.

**Main outcome measures:**

Six independent raters reassessed audio recordings for comparison with registry data. For calculation of agreement, the single measures intraclass correlation coefficient (ICC) was used for percentage of consonants correct (PCC) and non-oral speech errors, quadratic weighted kappa for velopharyngeal competence (VPC), and percentage agreement and kappa for quality indicators. The results of the three to four raters with the highest intra-rater and inter-rater reliability were used for comparison with registry data.

**Results:**

There was excellent agreement between registry data and reassessments for PCC (ICC, 0.93) and percentage of non-oral errors (ICC, 0.80). For VPC, one rater and registry data had good agreement (k, 0.704); the remaining cases had fair agreement (k, 0.476-0.554). The percentage agreement between registry data and reassessments for quality indicators ranged from fair to excellent. When calculated with kappa, agreement was good to excellent (mean of all k values, 0.67-0.70).

**Conclusions:**

The CLP registry variables PCC and percentage of non-oral errors and the quality indicators *without non-oral speech errors* and *competent/marginally incompetent velopharyngeal function* are reliable for use in clinical audits and research of 10-year-olds. The three-tier ratings of VPC have weaker reliability but can still be useful in more detailed analyses if interpreted with caution.

## Introduction

Auditory perceptual assessment is critical when evaluating the results of palatal surgery in patients with cleft palate and when making clinical treatment decisions. Speech is a perceptual phenomenon, and auditory perceptual speech assessment is considered the gold standard for speech assessment.^1^ The degree of agreement within and between assessors is a determinant of the usefulness of the results in cleft lip and palate (CLP) speech research, and it should be reported to allow for interpretation of the reliability of the results.^2,3^

Speech results are affected by the listeners’ internal standards, which vary among listeners.^4^ Therefore, training in perceptual assessment is necessary to achieve high reliability of speech results.^5–8^ In research, it is recommended to use multiple listeners who are trained as well as blinded speech-language pathologists (SLPs) specialized in cleft palate speech.^2,3^

Because clinical decisions are dependent on reliable speech results, there is a need for reliable methods of perceptual assessment. The International Consortium for Health Outcomes Measurement working group for cleft care has published a standard set of outcome measures for the comprehensive appraisal of cleft care.^9^ The use of this standard set was designed to be feasible even in low-income countries, and it contains measures that are important to the patient and comparable across languages.^9^ It includes clinician-reported,^10–12^ caregiver-reported,^13^ and patient-reported^14^ speech outcomes. One of the clinician-reported speech outcomes is the percentage of consonants correct (PCC), originally developed by Shriberg and Kwiatkowski^15^ to be used for assessment of conversational speech. However, PCC can be used on single-word material if the results are not related to the severity of involvement.^10^ The second clinician-reported speech outcome is velopharyngeal competence (VPC), which is an overall rating of VPC based on the impression of hypernasality, audible nasal air leakage, and reduced pressure on consonants. VPC is rated on a 3-point scale called the VPC-R, which contains the scale values competent, marginally incompetent, and incompetent.^12^ The VPC-R has been shown to be reasonably valid.^12^

The purpose of the Swedish CLP registry is to evaluate CLP treatment, facilitate improvement of treatment methods, and ensure consistent treatment. All six Swedish regional CLP centers are connected to the registry.^16^ Children born with CLP are followed up to adulthood, and data on treatment results are registered at 18 months and 5, 10, 16, and 19 years of age. The clinician-reported speech outcomes are PCC, percentage of non-oral speech errors, and VPC. The CLP registry data on these three outcomes at 5 years of age have been shown to be reliable.^17,18^ The variables have been dichotomized, and the binary quality indicators ≥*86% correct consonants* (corresponding to age-appropriate consonant production at 5 years of age), *without non-oral speech errors,* and *competent/marginally incompetent velopharyngeal function* are commonly used in the presentation of results.^19^ The Swedish CLP registry can be used for open comparisons of treatment results and provide a basis for improvements of treatment methods. Nevertheless, if deviating negative results are consistently observed at one CLP center, further analyses are needed before making changes to the treatment protocol.^19^

In an effort to maintain high reliability in the CLP registry speech data, we introduced listening calibration as a recurring item at the annual meeting of Swedish SLPs specialized in CLP speech. Additionally, calibration among SLPs within regional CLP teams occurs twice a year. Furthermore, we have designed guidelines with minimal requirements for SLPs registering speech data in the CLP registry. Introductory training must be completed, and participation in regional and national calibration is mandatory. The SLP must work with patients with CLP to the extent necessary to maintain competence.

Speech in individuals with cleft palate usually improves with age.^20,21^ It can be assumed that intra-rater and inter-rater agreement increase as speech improves. For example, in phonetic transcription, severe speech disorders have been associated with low transcriber agreement.^22^ However, because the data in the Swedish CLP registry are used for continuous open comparisons, it is important to ensure high reliability in speech data at the age of 10 years. Therefore, the present study was performed to assess the reliability of speech data and speech-related quality indicators in the CLP registry at 10 years of age. The research questions were as follows:

1. Are data on PCC, percentage of non-oral speech errors, and VPC at 10 years of age in the CLP registry reliable?
2. Are the quality indicators ≥*92.3% correct consonants* (corresponding to age-appropriate consonant production in 10-year-olds, defined as PCC of at least at −2 standard deviations from the mean in normative data^23^) and *without non-oral speech errors and competent/marginally incompetent velopharyngeal function* reliable for use at 10 years of age?

## Methods

### Participants

All children born in Sweden with non-syndromic unilateral CLP (UCLP) or bilateral CLP (BCLP) from 2009 to 2011 from all six Swedish CLP centers were included in this study. Children with missing recordings at 10 years (±12 months) of age or missing data on the variables PCC, percentage of non-oral speech errors, or VPC were excluded. This resulted in a total of 121 participating children (39 with BCLP, 82 with UCLP). In the group with BCLP, 15 were girls and 24 boys, and in the UCLP group 28 were girls and 54 boys. According to CLP registry data on VPC, 75 (62%) of the children had competent velopharyngeal function, 35 (28.9%) had marginally incompetent velopharyngeal function, and 11 (9.1%) had incompetent velopharyngeal function.

### Audio Recordings

All audio recordings were performed in a quiet room at one of the university hospitals participating in the study. The following equipment was used: an audio recorder (Zoom H4n; Zoom Corporation Hauppauge, NY, USA) (TASCAM HD-P2; TASCAM, Montebello, CA, USA) or a personal computer with Soundswell software (Saven Hitech AB, Stockholm, Sweden) in combination with a condenser microphone (Røde NT4; Røde Microphones, Sydney, Australia) (Sony ECM-MS957; Sony Corporation, Tokyo, Japan) (Pearl TL6C or Pearl CC3; Pearl Mikrofonlaboratorium AB, Åstorp, Sweden) (Sennheiser MKE 2 P-C; Sennheiser electronic GmbH & Co. KG, Wedemark, Germany). The participants read or named the words in the SVANTE single-word picture-naming test, of which the first 59 words are composed of oral consonants and the following five words are composed of nasal consonants.^23^ In addition, the participants repeated the SVANTE sentences, with the exception of seven cases in which the participants read the sentences and one case in which the sentences were missing. Eight sentences contained different high-pressure consonants (each sentence was loaded with the same consonant), two contained low-pressure consonants, one contained nasal consonants, and two contained transitions from nasals to stops. Continuous speech was also recorded, consisting of conversational speech, talking about a thematic picture, reading and re-telling of a text, or retelling of the Bus Story.^24,25^

### Data from the CLP Registry

Data registered at the 10-year assessment, based on the audio recordings, were retrieved from the CLP registry via Record Centre South (Lund, Sweden) and used for analysis. The speech data comprised the PCC, percentage of non-oral speech errors, and VPC.^23^ The assessments, audio recordings, and registrations for each participant were performed by an SLP who was part of the CLP team.

The target consonants in the single-word test were transcribed with semi-narrow transcription according to the International Phonetic Alphabet.^26^ Based on the transcriptions of target consonants in the first 59 words, PCC was calculated by dividing the number of correct consonants (if the target consonant was changed to another phoneme it was scored as incorrect) by the number of elicited consonants.^23^ The percentage of non-oral errors was calculated by dividing the number of non-oral errors (glottal and pharyngeal articulation as well as active nasal fricatives) by the number of elicited target consonants. VPC was rated on the VPC-R, the above-described 3-point scale containing the scale values “competent/sufficient,” “marginally incompetent/insufficient,” and “incompetent/insufficient” based on all the speech material of each participant.^23^ Refined guidelines for rating VPC were used. Marginally incompetent/insufficient was defined as having mild speech symptoms suggesting minor issues with the velopharyngeal closure, which may be sufficient in some contexts and speech materials but somewhat insufficient in others and would not lead to a recommendation about surgical treatment. Incompetent/insufficient was defined as having significant speech symptoms that, upon investigation with visualization of velopharyngeal function, would usually lead to a recommendation for surgical treatment. In cases of continuous glottal activity, VPC was rated as incompetent/insufficient because competent VPC was not demonstrated.

### Perceptual Reassessment and Analysis of Recordings

Unedited recordings were transferred to .wav files in the Soundswell software program (Saven Hitech AB). Twenty-four randomly chosen recordings were duplicated to assess the intra-rater agreement. One SLP from each of the six Swedish CLP centers reassessed the recordings. Their experience with cleft palate speech and participation in perceptual assessment for research purposes varied. Including PhD studies, SLP6 had 8.0 years of full-time experience and SLP5 had 2.2 years. SLP1, SLP2, and SLP3 had approximately 1.5 years of full-time experience. Although SLP4 had worked with CLP speech since 2009, she had only 0.9 years of full-time experience. Before the reassessment started, the SLPs calibrated themselves by individually assessing audio recordings of 7- and 10-year-olds with UCLP for 4 h. This was followed by a joint discussion of their assessments. Headphones were used for calibration and reassessment (AKG K271 MK II, AKG K240, or AKG K182; AKG Acoustics, Vienna, Austria) (Sony MDR-1AM2; Sony Corporation) (Creative Aurvana Live; Creative Technology Ltd., Jurong East, Singapore), and the recordings could be listened to repeatedly. The perceptual assessments and data analysis were carried out as described above.

### Speech-related Quality Indicators

Binary speech-related quality indicators are commonly used when reporting results from the Swedish CLP registry, and the reliability of these indicators was also assessed in the present study. The quality indicator *≥92.3% correct consonants* is based on PCC. The cut-off, 92.3%, corresponds to −2 standard deviations from the mean value in normative data of Swedish-speaking 10-year-olds without CLP.^23^ The second quality indicator, *without non-oral speech errors,* is based on the percentage of non-oral speech errors. To allow for a margin of error, up to 5% non-oral speech errors are permitted without being classified as non-oral errors. The third quality indicator, *competent/marginally incompetent velopharyngeal function*, is based on the VPC-R scale; specifically, the two first scale values are pooled.

### Statistical Analysis

Absolute agreement between and within SLPs, as well as between the data in the CLP registry and the reassessment results for PCC and percentage of non-oral speech errors, was calculated by the single measures intraclass correlation coefficient (ICC) with a two-way mixed-effects model. Agreement regarding VPC between and within SLPs, as well as between the data in the CLP registry and one independent listener at a time, was calculated using the quadratic weighted kappa. To assess inter-rater agreement among several SLPs at the same time, the mean of the kappa values from all pair-wise comparisons was calculated. Because the percentage agreement does not take chance agreement into account, this is an alternative method for assessment of inter-rater agreement involving several raters.^27^ The reliability of the quality indicators was calculated using point-by-point percentage agreement and Cohen's kappa, and the mean of the kappa values from all pair-wise comparisons was also calculated. The strength of agreement was interpreted according to Cicchetti^28^ (see Table 1).

**Table 1.** Interpretation of k Statistics, ICC, and Percentage Agreement According to Cicchetti.^28^

k or ICC	Observed agreement (%)	Levels of clinical or practical significance
<0.40	<70	Poor
0.40-0.59	70-79	Fair
0.60-0.74	80-89	Good
0.75-1.00	90-100	Excellent

k, kappa; ICC, intraclass correlation coefficient.

## Results

SLPs with poor or fair intra-rater and inter-rater agreement regarding a specific variable were excluded from further analysis of that variable.

### Reliability of PCC Reassessments

For PCC, the absolute intra-rater agreement as calculated by the single measures ICC was excellent for SLP2, SLP4, SLP5, and SLP6 (Table 2). Agreement was good for SLP1 and fair for SLP3 (Table 2). Thus, SLP3 was excluded from further analysis of PCC. For the remaining five SLPs (SLP1, SLP2, SLP4, SLP5, and SLP6), inter-rater agreement as calculated by the single measures ICC was excellent (ICC, 0.94; 95% confidence interval [CI], 0.92-0.96). For the binary quality indicator ≥*92.3% correct consonants*, the inter-rater agreement among the five SLPs assessed at the same time by point-by-point percentage agreement was fair (76%). After exclusion of SLP4, whose results were the most deviant from the other SLPs, the agreement increased to good (82%). Consequently, SLP4 was excluded from further analysis of PCC.

**Table 2.** Absolute Intra-rater Agreement as Calculated by Single Measures ICC for PCC and Percentage Non-oral Speech Errors and by Weighted Quadratic k for VPC.

	PCC		Non-oral errors		VPC	
	ICC	95% CI	ICC	95% CI	k	95% CI
SLP1	0.71	0.44-0.86	N/A	N/A	1	N/A
SLP2	0.83	0.61-0.92	.47	0.10-0.73	0.56	0.05-1
SLP3	0.58	0.23-0.79	.00	−0.38-0.39	0.58	0.03-1
SLP4	0.89	0.71-0.96	N/A	N/A	1	1-1
SLP5	0.86	0.70-0.93	N/A	N/A	0.63	0.15-1
SLP6	0.83	0.65-0.92	N/A	N/A	0.94	0.67-1

ICC: intraclass correlation coefficient; PCC: percentage of consonants correct; k: kappa; VPC: velopharyngeal competence; Non-oral errors: percentage of non-oral speech errors; CI: confidence interval; SLP: speech-language pathologist; N/A: not available.

### Reliability of Non-oral Speech Error Reassessments

SLP1, SLP4, SLP5, and SLP6 had scored no non-oral speech errors for the 24 participants whose recordings were used to assess intra-rater agreement. Thus, it was not possible to calculate absolute intra-rater agreement by the single measures ICC for these four SLPs for the percentage of non-oral speech errors (Table 2). For the two remaining SLPs (SLP2 and SLP3), intra-rater agreement was fair or poor (Table 2). The results of intra-rater agreement in the assessments of non-oral speech errors led us to exclude SLP2 and SLP3 from further analysis of the percentage of non-oral speech errors. Inter-rater agreement regarding the percentage of non-oral speech errors for SLP1, SLP4, SLP5, and SLP6 as calculated by the single measures ICC was excellent (ICC, 0.90; 95% CI, 0.87-0.92). Inter-rater agreement regarding the binary quality indicator *without non-oral speech errors* for SLP1, SLP4, SLP5, and SLP6, as assessed at the same time by point-by-point percentage agreement, was excellent (93%).

### Reliability of Velopharyngeal Competence Reassessments

Intra-rater agreement regarding VPC, calculated with the quadratic weighted kappa, was excellent for SLP1, SLP4, and SLP6 and good for SLP5 (Table 2). SLP2 and SLP3 had only fair intra-rater agreement for VPC and were therefore excluded from further analysis of VPC. Inter-rater agreement for VPC was calculated for two raters at a time with the quadratic weighted kappa for the four SLPs who had good or excellent intra-rater agreement (SLP1, SLP4, SLP5, and SLP6) (Table 3). The kappa values were good in three cases (SLP1/SLP5, SLP4/SLP5, and SLP5/SLP6) but only fair in three other cases (SLP1/SLP4, SLP1/SLP6, and SLP4/SLP6). The mean of all kappa values was fair. When the SLP with the most deviant assessment (SLP4) was excluded, the mean of all kappa values slightly increased to just above the limit for good (Table 3). SLP4 was excluded from further analysis of VPC. Among the remaining SLPs, inter-rater agreement regarding the binary quality indicator *competent/marginally incompetent velopharyngeal function* as assessed at the same time by point-by-point percentage agreement was excellent (90%).

**Table 3.** Inter-Rater Agreement for Velopharygeal Competence as Calculated by Quadratic Weighted k for the Four SLPs with the Best Intra-Rater Agreement, and Comparisons with Data from the Swedish CR.

	k	95% CI
SLP1/SLP4	0.51	0.35-0.67
SLP1/SLP5	0.62	0.46-0.79
SLP1/SLP6	0.56	0.37-0.75
SLP4/SLP5	0.66	0.50-0.83
SLP4/SLP6	0.52	0.38-0.66
SLP5/SLP6	0.67	0.50-0.85
Mean	0.59	0.43-0.76
	k	95% CI
SLP1/SLP5	0.62	0.46-0.79
SLP1/SLP6	0.56	0.37-0.75
SLP5/SLP6	0.67	0.50-0.85
Mean	0.62	0.44-0.80
	k	95% CI
SLP1/SLP5	0.62	0.46-0.79
SLP1/SLP6	0.56	0.37-0.75
SLP5/SLP6	0.67	0.50-0.85
SLP1/CR	0.48	0.30-0.65
SLP5/CR	0.55	0.38-0.73
SLP6/CR	0.70	0.55-0.86
Mean	0.60	0.42-0.77

k, kappa; SLP, speech-language pathologist; CR, cleft lip and palate registry; CI, confidence interval

### Comparison of Registry Data and Data from Reassessments

For PCC, agreement between the data in the CLP registry and the values obtained from reassessments by SLP1, SLP2, SLP5, and SLP6, calculated by the single measures ICC, was excellent (ICC, 0.93; 95% CI, 0.91-0.95). For the percentage of non-oral errors, agreement between the data in the CLP registry and the values obtained from reassessments by SLP1, SLP4, SLP5, and SLP6 was also excellent (ICC, 0.80; 95% CI, 0.75-0.85).

The agreement between VPC data in the Swedish quality registry for CLP and reassessments by SLP1, SLP5, and SLP6, as calculated with the quadratic weighted kappa, was also assessed (Table 3). The agreement between the CLP registry and SLP6 was good; however, it was fair in the other cases. The mean of the kappa values was just below the limit for good (0.598); when rounded up to two decimals (0.60), it corresponded to good agreement (Table 3).

### Speech-related Quality Indicators

For the quality indicator *≥92.3% correct consonants*, there was 71% agreement between SLP1, SLP2, SLP5, SLP6, and the CLP registry data (compared at the same time) in assessing whether the children were above or below the cut-off of 92.3% correct consonants. This corresponded to fair agreement. Calculation of the mean of the kappa values from all pair-wise comparisons resulted in good agreement, with the lower limit of the 95% CI within the interval of fair agreement and the upper limit within the interval of excellent agreement (Table 4).

**Table 4.** Agreement for the Speech-related Quality Indicators in Pair-wise Comparisons of Reassessments by SLPs with the Best Intra-rater and Inter-rater Agreement and Data from the Swedish CR as Calculated by Percentage Agreement and Cohen's k.

≥92.3% correct consonants			
	%	k	95% CI
SLP1/SLP2	92	0.77	0.63-0.90
SLP1/SLP5	89	0.70	0.55-0.85
SLP1/SLP6	87	0.66	0.51-0.81
SLP2/SLP5	93	0.84	0.73-0.96
SLP2/SLP6	89	0.75	0.62-0.88
SLP5/SLP6	91	0.77	0.65-0.90
SLP1/CR	79	0.49	0.32-0.66
SLP2/CR	84	0.62	0.47-0.77
SLP5/CR	83	0.60	0.45-0.76
SLP6/CR	79	0.52	0.36-0.69
Mean	–	0.67	0.53-0.82

Without non-oral speech errors			
	%	k	95% CI
SLP1/SLP4	97	0.85	0.70-0.99
SLP1/SLP5	96	0.79	0.62-0.97
SLP1/SLP6	95	0.74	0.55-0.94
SLP4/SLP5	98	0.87	0.72-1
SLP4/SLP6	97	0.82	0.64-0.99
SLP5/SLP6	99	0.95	0.85-1
SLP1/CR	93	0.63	0.40-0.85
SLP4/CR	94	0.69	0.47-0.90
SLP5/CR	97	0.80	0.61-0.99
SLP6/CR	98	0.84	0.67-1
Mean	–	0.80	0.62-0.96

Competent/marginally incompetent velopharyngeal function			
	%	k	95% CI
SLP1/SLP5	92	0.66	0.46-0.85
SLP1/SLP6	92	0.62	0.41-0.83
SLP5/SLP6	97	0.83	0.66-0.99
SLP1/CR	90	0.55	0.36-0.73
SLP5/CR	95	0.74	0.55-0.94
SLP6/CR	97	0.80	0.61-0.99
Mean	–	0.70	0.51-0.89

SLP, speech-language pathologist; CR, cleft lip and palate registry; k, kappa; CI, confidence interval.

For the indicator *without non-oral speech errors*, there was 92% agreement between SLP1, SLP4, SLP5, SLP6, and the CLP registry data (compared at the same time), corresponding to excellent agreement. Furthermore, the mean of the kappa values from all pair-wise comparisons was within the category of excellent agreement (Table 4).

For the indicator *competent/marginally incompetent velopharyngeal function*, there was 88% agreement between SLP1, SLP5, SLP6, and the CLP registry data (compared at the same time), corresponding to good agreement. The mean of the kappa values from all pair-wise comparisons also indicated good agreement (Table 4).

## Discussion

The first aim of this study was to assess the reliability of data on speech variables in the CLP registry at 10 years of age. We found that PCC and percentage of non-oral speech errors were reliable measures, with excellent agreement between the CLP registry and reassessments. For the VPC ratings, however, agreement between the majority of the raters and the CLP registry was only fair, with only one rater reaching good agreement with the CLP registry.

The second aim was to assess the reliability of speech-related quality indicators in the CLP registry at 10 years of age. Agreement between the CLP registry and reassessments for the quality indicators varied from good to excellent. All three quality indicators (*without non-oral speech errors*, *competent/marginally incompetent velopharyngeal function*, and *≥92.3% correct consonants*) can therefore be considered reliable.

### Interpretation of Results in Relation to Previous Studies

For PCC, percentage of non-oral errors, and the quality indicators *without non-oral speech errors* and *competent/marginally incompetent velopharyngeal function*, the reliability of the registry data at 10 years of age found in the present study was comparable to that at 5 years of age in previous studies.^17,18^ For the 3-point rating of VPC and the binary quality indicator based on PCC, however, the reliability was lower at 10 years of age in the present study than at 5 years of age in previous studies.^17,18^

A possible contributing factor to the lower levels of agreement in the present study than in previous studies is that the 10-year-old participants had, as expected,^20,21^ better speech results than the 5-year-old participants, leading to low variance in the speech results. When reviewing the CLP registry data in our study and in a previous study,^17^ 9% of the 10-year-olds had incompetent velopharyngeal function compared with 20% of the 5-year-olds. Lower variance requires higher levels of agreement between raters to achieve adequate inter-rater reliability.^29^ For non-oral speech errors, the rate of errors in the recordings was so low that it was judged to invalidate the calculation of intra-rater agreement.

### Experience of Raters

The raters in this study were SLPs specialized in cleft palate speech. However, the median duration of full-time experience in rating cleft palate speech was only 1.5 years, and only one rater had more than 3 years of full-time experience. The inclusion of several raters with limited experience was an active choice because it accurately reflects most clinical settings. Because CLP is a relatively rare condition,^30^ it takes time for new clinicians to gain experience in CLP care. Sweden is sparsely populated relative to many other countries and consequently has an especially small patient population with CLP (approximately 175 newborns each year),^19^ and the catchment areas of individual CLP centers are geographically large. However, a previous study revealed no negative impacts on the surgical treatment results in treatment centers with lower patient volumes.^19^ This might be explained by the fact that some surgeons in those centers participate in foreign exchanges to increase their surgical experience. For SLPs working in CLP centers with low patient volume, participating in perceptual evaluations for research (such as the present study) is one way to increase experience of perceptual speech assessment. This will lead to improved intra-rater and inter-rater reliability.

In the Americleft Speech Project, Chapman et al.^6^ compared inter-rater reliability before and after training sessions and found higher agreement after training for hypernasality but only moderate improvement for audible nasal emission. Ratings of VPC are based in part on perceived hypernasality, audible nasal emission, and reduced pressure on consonants; therefore, increasing the amount of structured training ahead of perceptual calibrations may or may not improve rater agreement for VPC scores.

When screening the scores of individual SLPs, we found that less experienced SLPs tended to make stricter judgments compared with the data in the CLP registry, whereas the judgments of the most experienced SLP were generally in line with the registry data. This might have occurred because less experienced SLPs make great efforts to avoid missing any speech abnormalities when participating in a research study and therefore tend to make stricter assessments, whereas more experienced SLPs with more stable internal standards for perceptual speech assessment tend to make equivalent assessments in clinical practice and in research projects.^31^

### Exclusion of Raters

Based on the intra-rater reliability scores, one SLP was excluded from the comparisons with the registry data regarding PCC. Additionally, one SLP with deviant results for the quality indicator ≥*92.3% correct consonants* was excluded from further comparisons of the registry data regarding PCC. Because of insufficient intra-rater reliability, two SLPs were excluded from comparisons with the registry data regarding non-oral errors. Two SLPs were excluded from comparisons with the registry data regarding VPC because of insufficient intra-rater reliability, and one SLP was excluded because of deviant VPC results to increase inter-rater reliability. This resulted in the CLP registry data being compared to reassessments of only four of the six SLPs for PCC, four for non-oral errors, and three for VPC. Excluding raters with poor to fair intra-rater reliability was considered necessary to ensure reproducibility of the results.^1^ SLP4 was excluded from the comparisons with the registry data regarding PCC and VPC because the results indicated that the internal standards of SLP4 deviated from those of the other raters.^6^

### Clinical Implications

While this study was focused on the reliability of speech data in the CLP registry, the results also illustrate another important point: the reliability of speech data is as dependent on individual raters’ experience^32^ and internal standards^33^ as on speech materials and samples.^34^ Consequently, the reliability of speech results, once proven, cannot be assumed to remain constant without continuous efforts to calibrate raters’ internal standards. As pointed out by Lohmander and Olsson^2^ and Sell,^3^ research based on perceptual speech analysis must report both inter-rater and intra-rater reliability for the results to be repeatable and generalizable.

## Conclusion

The results of this study indicate that the CLP registry data on PCC and percentage non-oral speech errors as well as the quality indicators *≥92.3% correct consonants*, *without non-oral speech errors*, and *competent/marginally incompetent velopharyngeal function* at the age of 10 years have sufficient reliability and are suitable for use in clinical audits and research. Three-tier ratings of VPC in the same population at the age of 10 years have weaker reliability but can still be useful in more detailed analyses if interpreted with caution.
